# BB0347, from the Lyme Disease Spirochete *Borrelia burgdorferi*, Is Surface Exposed and Interacts with the CS1 Heparin-Binding Domain of Human Fibronectin

**DOI:** 10.1371/journal.pone.0075643

**Published:** 2013-09-27

**Authors:** Robert A. Gaultney, Tammy Gonzalez, Angela M. Floden, Catherine A. Brissette

**Affiliations:** Department of Microbiology and Immunology, University of North Dakota School of Medicine and Health Sciences, Edwin C. James Medical Research Facility Grand Forks, North Dakota, United States of America; University of Toledo School of Medicine, United States of America

## Abstract

The causative agent of Lyme disease, *Borrelia burgdorferi*, codes for several known fibronectin-binding proteins. Fibronectin a common the target of diverse bacterial pathogens, and has been shown to be essential in allowing for the development of certain disease states. Another borrelial protein, BB0347, has sequence similarity with these other known fibronectin-binding proteins, and may be important in Lyme disease pathogenesis. Herein, we perform an initial characterization of BB0347 via the use of molecular and biochemical techniques. We found that BB0347 is expressed, produced, and presented on the outer surface of intact *B. burgdorferi*. We also demonstrate that BB0347 has the potential to be important in Lyme disease progression, and have begun to characterize the nature of the interaction between human fibronectin and this bacterial protein. Further work is needed to define the role of this protein in the borrelial infection process.

## Introduction

*Borrelia burgdorferi* is a pathogenic spirochete endemic to North America and the causative agent of Lyme disease. The etiology of the disease is unique, often resulting in a bulls-eye shaped rash surrounding the area of infection [1]. In untreated patients, further complications can arise, including carditis, arthritis, and neuroinflammation, with a potential for chronic symptoms [2]. A current focus in *B. burgdorferi* research is to identify which factors allow the bacterium to evade the host immune response in the patients presenting such symptoms [3].

The spirochete is spread by ticks of the genus *Ixodes* [1], which are found throughout large areas of the United States [4]. The number of Lyme disease diagnoses in the US exceeds 30,000 per year–making this the most prevalent arthropod-borne infection in the US [5]–and there is evidence that recent changes in the climate [6] as well as current forestry practices [7], [8] are contributing to an increase in the number of annual occurrences of Lyme disease in humans.

It is well known that infectious organisms such as bacteria often depend on adherence to host tissues or cells in order to cause disease [9]–[13]. The human extracellular matrix (ECM) is an ideal candidate for this binding as it contains a number of molecules with which pathogens, including *Borrelia burgdorferi* [14], [15] can interact. One of these molecules, the glycoprotein fibronectin (Fn), is found ubiquitously in human tissues and also at very high concentrations as a soluble form in plasma [16]. Importantly, the Arg-Gly-Asp (RGD) motif found in the cell-binding domain of Fn allows this protein to interact with the α_5_β_1_ integrin found in the plasma membrane of host cells [17]. *Borrelia burgdorferi* has several currently identified proteins that can bind to human fibronectin: BBK32 [18], RevA, RevB [19], CRASP-1 [20], and the putative gene product BB0347 [21].

The role of fibronectin-binding phenomena in *Borrelia* is difficult to elucidate due to this apparent redundancy in Fn-recognition proteins. Mutants lacking BBK32 exhibit reduced infectivity, and BBK32 has recently been shown to mediate vascular adhesion *in vivo* [21]–[24]. However, a definitive role or mechanism for the full complement of Fn-binding proteins in *B. burgdorferi* remains elusive. However, Fn-binding phenomena are well studied in a number of other pathogens [25] including the opportunistic pathogen *Staphylococcus aureus* [26]–[29]. This bacterium is a major cause of nosocomial infections and can have a high mortality rate; especially in strains that have become resistant to antibiotic treatment [29]. The role of fibronectin binding in pathogenesis is particularly evident in *S. aureus* as deletion of the two known Fn binding proteins (FnBPA and FnBPB) in this pathogen can significantly attenuate virulence in a mouse model [30].

FnBPA is a well-characterized protein. It interacts with fibronectin by a series of eleven C-terminal Fn-binding domains [31]. Fibronectin bound by FnBPA has an RGD sequence accessible by the aforementioned α_5_β_1_ integrin complex on the surface of host cells, which allows for uptake of the intact *S. aureus* bacterium [26]. The pathogen is postulated to internalize as an immune evasion strategy [26], [27]. Nearly all clinical isolates of *S. aureus* present one of the two known Fn-binding proteins [31], and inert latex beads coated with FnBPA are able to be taken up by non-phagocytic cells in the presence of fibronectin [32]; further highlighting the importance of these Fn-binding proteins in the infectious life cycle of *S. aureus*, as well as the potential importance for Fn-recognition and binding in other pathogenic bacteria. Some evidence suggests that *B. burgdorferi* is able to also be endocytosed by host cells [33], [34], and that this phenomenon may depend on a Fn-binding protein [35]. However, no role was found for the well-characterized borrelial protein BBK32 in this process.

To continue the research into the role of fibronectin interactions in Lyme disease etiology, we began work on the putative BB0347 gene product, which has been shown to bind Fn previously [21]. Additionally, this gene/protein has been examined cursorily in previous papers as a control for transposon mutagenesis [36] and as having a peptide homologous to the OspA T-cell activating epitope OspA_165–173_ [37]. However, no work has been done to verify that the BB0347-Fn interaction is functional in live spirochetes. Herein, we begin to characterize the expression of *bb0347* as well as the interaction of the protein with human Fn to facilitate better understanding of the role of BB0347 in human disease.

## Materials and Methods

### Animals and Ethics Statement

The protocol for animal infection was approved by the University of North Dakota Institutional Animal Care and Use Committee (IACUC protocol #1101-2), in accordance with Association for Assessment and Accreditation of Laboratory Animal Care guidelines (Animal Welfare Assurance: A3917-01). Mice used for this study were 4 to 6 week-old female C3H/HeNHsd (Harlan; Madison, WI), and were cared for under the guidelines of the National Research Council of the National Academics *Guide for the Care and Use of Laboratory Animals* (8th Edition). All efforts were made to minimize animal suffering.

### Bacteria and Culture Conditions

*Borrelia burgdorferi* strain MI-16, an infectious clone of the sequenced type strain [38] that contains all parental plasmids [39], was used for all experiments at low passage levels. The spirochetes were grown in modified Barbour-Stoenner-Kelly (BSK II) medium [40] supplemented with 6% rabbit serum (Pel-Freeze, #A00274; Rogers, AR) at 23 or 34°C to cell densities of approximately 1×10^7^ cells/mL, i.e. mid-log phase (except where otherwise noted). For infection studies, the spirochetes were confirmed, via multiplex PCR, to possess all virulence plasmids prior to infection as described by Bunikis et al. [41]. Additionally, One Shot TOP10 *Escherichia coli* (Life Technologies, #C4040-06; Grand Island, NY) and chloramphenicol-resistant Rosetta(DE3)pLysS *E. coli* (Novagen, #71401-4; Madison, WI) strains were used in cloning and production of recombinant proteins, respectively.

### Recombinant Protein Production

DNA sequences were amplified via PCR using DeepVent DNA Polymerase (NEB, #M0258S; Ipswich, MA) to prevent addition of the 3′ A-overhang onto amplified nucleotide segments. Primers used for amplification are listed in Table 1. The nucleotide sequences were then cloned into the pET200 expression vector (Life Technologies, #K200-01), transformed into TOP10 *E. coli,* and cells were plated on LB agar supplemented with kanamycin (50 µg/mL–MP Biomedicals, # 0215002925; Santa Ana, CA). Resulting colonies were screened by colony PCR for the appropriately sized insert and further verified by bidirectional sequencing of purified plasmid DNA (Davis Sequencing; Davis, CA).

**Table 1:** Primers used in this study.

Name	Sequence (5′–3′)		Use
Fla3*		GGGTCTCAAGCGTCTTGG	Amplification of *flaB* for QRT-PCR
Fla4*		GAACCGGTGCAGCCTGAG	
CAB105		ACATTGAAAACGAAAAGGAA	Amplification of *bb0347* for QRT-PCR
CAB106		AAAGAATTTTTGCCCTTTTT	
BB0347protF		CACCATGATAAAAATGTCTTTGAATTACACTG	Cloning of BB0347 into pET200
BB0347protR		TTAGGTTTGATTTTTTATTTTTTTTATTAG	
OspCprotF		CACCTGTAATAATTCAGGGAAAG	Cloning of OspC into pET200
OspCprotR		TTAAGGTTTTTTTGGACTTTCTG	
FlaBprotF		CACCATTATCAATCATAATACATCAGCTA	Cloning of FlaB into pET200
FlaBprotR		TTATCTAAGCAATGACAATGACAAAACATATTGG	

* All primers were designed during the course of this study, except Fla3 & 4, which have been described previously [44].

To produce proteins, plasmids with appropriately inserted coding regions were transformed into Rosetta(DE3)pLysS and plated on LB agar with kanamycin (50 µg/mL) and chloramphenicol (30 µg/mL–Sigma, #C-0378). Individual colonies were selected and inoculated into either super broth (SB–32 g Tryptone, 20 g yeast extract, 10 g NaCl, per liter of H_2_O) starter culture or Dual Media Set (Zymo, #M3011; Irvine, CA) Expansion broth (EB) overnight, both supplemented with kanamycin and chloramphenicol as above. SB starter culture was transferred (≤1∶50 dilution) into final cultures of antibiotic-supplemented SB and allowed to grow to an OD ∼0.5, then induced with 0.1–0.3 mM isopropyl β-D-1-thiogalactopyranoside (IPTG) for four hours. EB was transferred to antibiotic-supplemented Dual Media Set Overexpression Broth and allowed to grow overnight at 37°C with shaking at approximately 200 rpm. From both media, the cells were spun down, re-suspended in MagneHis wash buffer (MHWB-100 mM HEPES, 10 mM imidazole), and lysed via sonication with a Model 705 Sonic Dismembrator (Fisher Scientific; Pittsburg, PA). The recombinant proteins were purified with Nickel bead affinity chromatography using MagneHis Ni-Particles (Promega, #V8565; Madison, WI) and magnetic stands. Four washes using MHWB were used to remove contaminating proteins, and MagneHis elution buffer (100 mM HEPES, 500 mM imidazole) was used to elute the purified recombinant proteins from the Nickel particles. This final elution buffer was replaced with phosphate buffered saline via dialysis in 3,500 MWCO dialysis cartridges (Pierce, #66330; Rockford, IL). Final protein concentrations were determined by a BCA assay (Pierce, #23227) and purities were assessed by SDS-PAGE followed by staining with Coomassie brilliant blue dye.

### Overlap PCR

To generate an N-terminal truncation of rBB0347, an overlap PCR was performed with the primers listed in Table 1. The Expand High Fidelity PCR system (Roche, #11732641001; Indianapolis, IN) was used with the following thermal cycler protocols: 94°C-3 min; (94°C-30 sec, 50°C-30 sec, 67°C-6 min)×10; (94°C-30 sec, 50°C-30 sec, 67°C-6 min with Δ15 sec each cycle)×25; and a final extension of 7 min at 67°C. Following the PCR, the product was run on an agarose gel and purified with a Qiaquick gel extraction kit (Qiagen, #28704; Valencia, CA). Following purification, the DNA was digested with DpnI (NEB, R0176S) overnight at a concentration of 400 U/mL to ensure that no parent DNA remained, after which point, plasmid was transformed into One Shot TOP10 *E. coli* as above. All other steps for verification and protein production were performed as detailed above.

### Fibronectin-binding ELISAs

1.0 µg of various proteins in carbonite coating buffer (0.32 g Na_2_CO_3_, 0.586 g NaHCO_3_ per 200 mL H_2_O) were coated overnight at 4°C on NUNC Maxisorp 96-well plates (Thermo, #T-3020-2; Rochester, NY). The next day, the plates were blocked with 300 µL SEA BLOCK blocking buffer (Thermo, #37527), and treated with 100 µL of varying concentrations of Fn (as listed in each figure) or rBB0347 (FnHBD assay) at 37°C for 1.5 hours. 100 µL of rabbit-αFn antibodies (Sigma, #F3648; St. Louis, MO) were employed at a 1∶500 dilution in PBS to determine the level of Fn binding. Alternatively, for the FnHBD assay, rabbit polyclonal-αBB0347 (Proteintech, Chicago, IL) was used at a 1∶1000 dilution. Goat αRabbit-IgG antibodies conjugated to horseradish peroxidase (Pierce, #31460) were then used at a 1∶5000 dilution in PBS for detection (100 µL). All previous steps, including the protein coating, were followed by three washes with PBS supplemented with 0.05% (v/v) Tween-20. Next, as a substrate, 100 µl One Step Turbo TMB (Thermo, #34022) was added per well, and the reaction was stopped via addition of an equal volume of 2 N H_2_SO_4_. Absorbance was read at 450 nm with an Epoch spectrophotometer with Gen5 data analysis software (BioTek; Winooski, VT). K_D_ was determined as the concentration of added protein at half-maximal binding, assuming saturation, as described previously [19].

### BB0347-Fn Inhibition Assays

ELISAs were performed as described above, but with minor modifications. The PBS solution containing the Fn was supplemented with excess NaCl (0–600 mM), Heparin (0–2 mM–Calbiochem, #374858; San Diego, CA) or ε-aminocaproic acid (0–300 mM–Sigma, #A7824). Alternatively, 96-well plates were coated as above with 0.5 µg rBB0347 or rTP0483 [42] and an inhibition assay was performed using Fn cell-binding domain-specific antibodies (Univ. of Iowa DSHB, #P1H11) supplemented to a concentration of either 0 or 2.5 µg/mL with 5 µg/mL of human Fn in PBS. Binding was analyzed as detailed previously, starting with detection of Fn-binding with the αFn polyclonal antibodies. Binding was normalized to the 0 µg/mL inhibitor level for each of the ELISAs.

### Western Blotting

Samples were run on a 12.5% SDS-PAGE and blotted onto a nitrocellulose membrane. The membranes were blocked overnight at 4°C with 5% non-fat dry milk in Tris-buffered saline supplemented with 0.1% Tween (TBST). Various primary antibodies or sera were used as indicated in the text, and HRP-conjugated secondary antibodies directed against IgG from the primary antibody host were employed. Extensive washing with TBST followed each step. SuperSignalWest Pico chemiluminescent substrate (Thermo, #34080) acted as the substrate for all of the experiments performed herein. Luminescence was read with via a Biochemi cooled camera and Epichemi^3^ darkroom with LabWorks 4.5 software (UVP; Upland, CA).

### Protease Protection Assay

Spirochetes were grown in culture until optimal production of BB0347 was observed (late log phase). Cells were then harvested by centrifugation at 4000×g for 20 minutes and washed once with PBS supplemented with 10 mM MgCl_2_ to remove media components; then centrifuged again. Bacteria were counted and re-suspended in the same buffer at a density of 2×10^9^ cells/ml. These bacteria were treated for various times with PBS (as a negative control), proteinase K (40 µg/mL), pronase (0.05 µg/mL), and trypsin (40 µg/mL) at 34°C. The reactions were stopped via the addition of EDTA (1.0 mM final–pronase only), PMSF (Sigma, #93482) (800 µM–all), and Pefabloc SC (Roche, #11585916001) (0.25 mg/mL–pronase and trypsin), and subsequent incubation on ice for 10 min. After this time, the cells were spun down and re-suspended in SDS-PAGE Sample loading buffer and boiled for 10 minutes to lyse the cells. A Western blot was performed as described above. Protein levels were determined with polyclonal rabbit antibodies against BB0347, FlaB (negative control), and OspC (positive control). All three of these antibodies were produced by Proteintech (Chicago, IL) using recombinant proteins produced in our laboratory as previously [43].

### QRT-PCR

*Borrelia burgdorferi* was grown at two different temperatures to mid-log phase as described above, then spun down at 4°C for 10 min at 6000×g and washed three times with PBS with a spin following each wash. Total RNA was isolated from each condition using the Trizol reagent (Ambion, #15596-018; Foster City, CA), and cDNA was synthesized using the SuperScript III first strand synthesis system (Life Technologies, #18080-051). Primers used for the QRT-PCR were Fla3 and Fla4 [44] to amplify *flaB* mRNA as a control and CAB105 and 106 to amplify *bb0347* mRNA (Table 1). PCR was performed on a MyiQ2 Thermal Cycler with dedicated software (Bio-Rad; Hercules, CA) and the run parameters were 95°C-3 min, (95°C-30 sec, 48°C-30 sec)×40, 95°C-1 min, and, finally, a melt curve starting at 45°C with Δ0.5°C in 10 sec intervals. Each reaction included a sample that lacked template and a sample of RNA processed without reverse transcriptase to test for DNA contamination of reagents.

### Immunofluorescence Microscopy

Bacteria were grown and centrifuged as in the protease protection assay protocol, except that two washes were performed in non-supplemented PBS. Bacteria were concentrated 50-fold in the final suspension, into which slides were placed for 15 min at room temperature to allow for adherence of spirochetes. Without allowing time for drying, the bacteria were fixed to the slide with a 10% formalin solution (Ricca Chemical, #3190-5; Arlington, TX) for 15 minutes. To confirm effectiveness of our αFlaB antibody, some spirochete membranes were disrupted by desiccation prior to formalin fixation. After treatment, the bacteria were washed 3 times with PBS and then blocked with 1.0% BSA in PBS overnight at 4°C with gentle agitation. The following day, the slides were washed again with PBS, and treated with affinity-purified antibodies against FlaB (1∶1000 dilution in PBS), OspC (1∶500), or BB0347 (1∶50) for two hours at room temperature, followed by a 1.5-hour incubation with anti-rabbit IgG conjugated to Dylight 488 (KPL, #072-03-15-06; Gaithersburg, Maryland). Slides were then mounted with Vectashield mounting medium supplemented with 4′, 6-diamidino-2-phenylindole (DAPI) (Vector, #H-1200; Burlingame, CA). Microscopy was carried out on an Olympus BX51 microscope with a DP71 Camera and Cellsens standard viewing software (Olympus; Center Valley, PA). Fluorescent percentages were determined by dividing the number of fluorescent cells for both OspC and BB0347 by the total number of DAPI- stained spirochetes. The number given for each protein is the average of at least 5 different fields of view from several slides.

### Immunogenicity of BB0347

Mice were infected by needle inoculation with 10^6^ cells of *B. burgdorferi* MI-16. Sera were harvested from the mice pre-inoculation and eight weeks post-inoculation. Purified rBB0347ΔN53 was run on an SDS-PAGE gel and transferred to a nitrocellulose membrane, and a Western blot was performed using a 1∶500 dilution of the harvested serum as the source of primary antibodies. αMouse-IgG (GE Healthcare, #NA931; Waukesha, WI) was used to detect any native αBB0347 antibodies from the infected mice, and substrate was used as described above. As a control, the purified αBB0347 affinity purified antibodies were used, and these were recognized with Goat αRabbit-IgG previously used in the ELISAs and protease protection assays. All other steps are detailed in the Western blotting section.

We determined antibody titers as previously [43]. Briefly, the same sera used previously were serially diluted tenfold and used to detect rBB0347 coated onto ELISA plates at a concentration of 10 µg/mL. To detect specific binding, values were compared to those of uninfected mouse serum.

### Statistical and Computational Analyses

Statistical analysis was carried out using either a two-tailed Student’s t-test assuming unequal variance, or One-way Analysis of Variance (ANOVA) followed by a Tukey’s *post hoc* test, when multiple comparisons were made. (*: p<0.05, **: p<0.01, ns: not statistically significant for all figures). Computational analyses, including CLUSTAL alignment, were carried out using the default parameters, and programs were accessed through the SDSC Biology Workbench (workbench.sdsc.edu).

## Results

### Borrelia Burgdorferi Expresses bb0347

**Figure 1:**
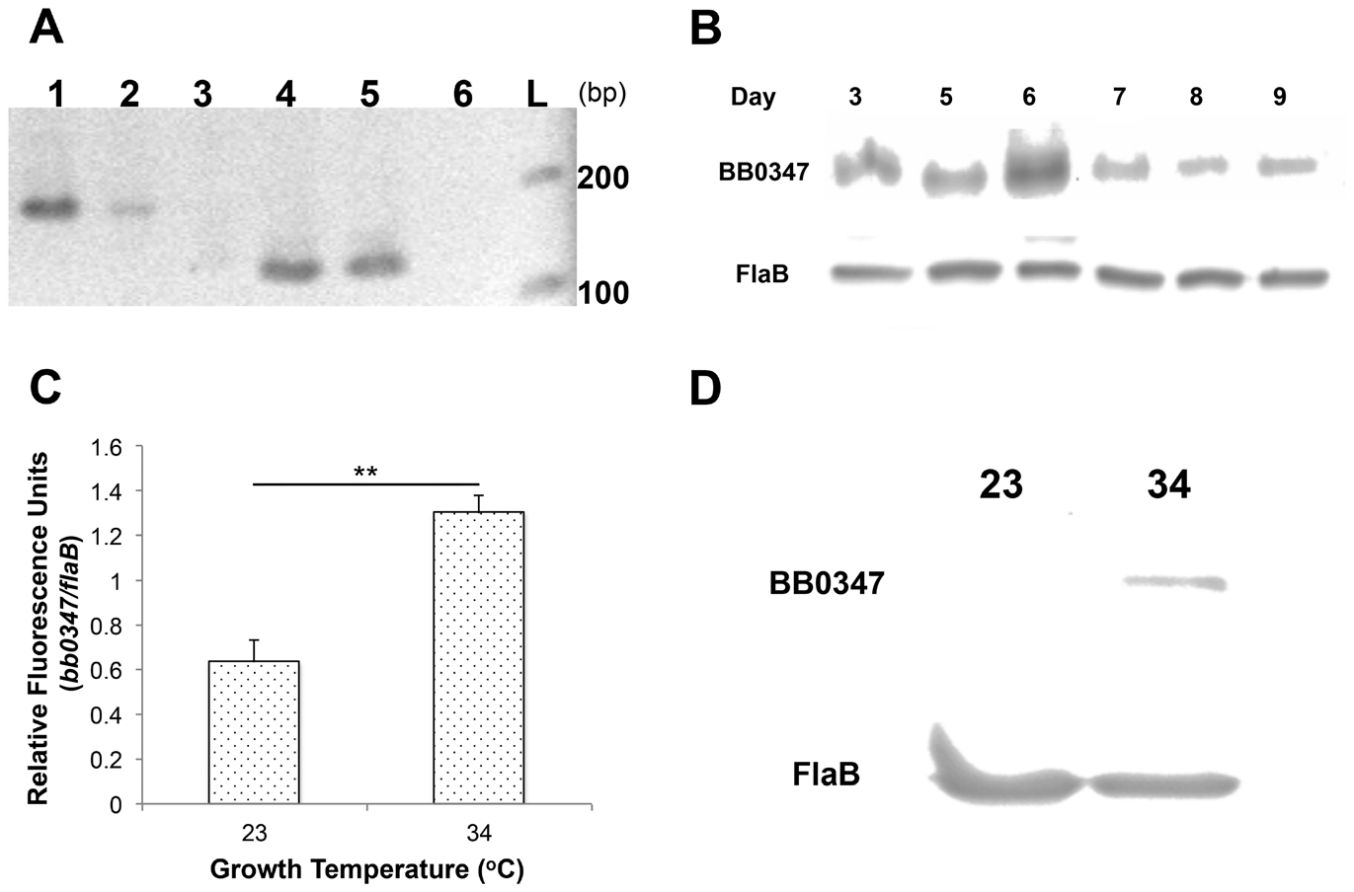
BB0347 is expressed and produced in culture. **A)** Expression of BB0347 was verified by RT- PCR with *flaB* mRNA as a control. Lane 1: *flaB* from genomic DNA, lane 2: *flaB* from cDNA, lane 3: no RT control, lane 4: *bb0347* from genomic DNA, lane 5: *bb0347* from cDNA, lane 6: no RT control, L: ladder. **B)** Western blotting shows that BB0347 protein is produced in the spirochete at all sampled time points. **C)** QRT- PCR of *bb0347* at two different temperatures of incubation with *flaB* as a standard. **D)** Western blot using αBB0347 and αFlaB against whole-cell lysates from spirochetes grown at either 34 or 23°C to similar cellular densities. All figures are representative of at least two independent experiments with similar results, and error bars indicate ±SEM.

Although a BLAST search shows that the *bb0347* gene product has putative Fn-binding domains (data not shown), and recombinant BB0347 has been cursorily examined [21], the actual expression of the gene *in vivo* has not been verified. We used RT-PCR to confirm that *bb0347* is actively expressed (i.e. not a pseudogene [45]–[48]) in *B. burgdorferi* (Fig. 1A) using primers listed in Table 1. Once this expression was verified, we ensured that BB0347 protein was also produced by *B. burgdorferi* MI-16 *in vitro* at every day sampled (Fig. 1B). No consistent differences were seen between the different days of incubation, and the data presented are only indicative of a single culture over time. Additionally, *bb0347* mRNA was present at higher levels when the bacterium was grown at 34°C than when cultured at 23°C (Fig. 1C). This finding was supported by comparing BB0347 protein levels at the same two temperatures by Western blot (Fig. 1D).

### *In vitro* Binding of Fibronectin

**Figure 2:**
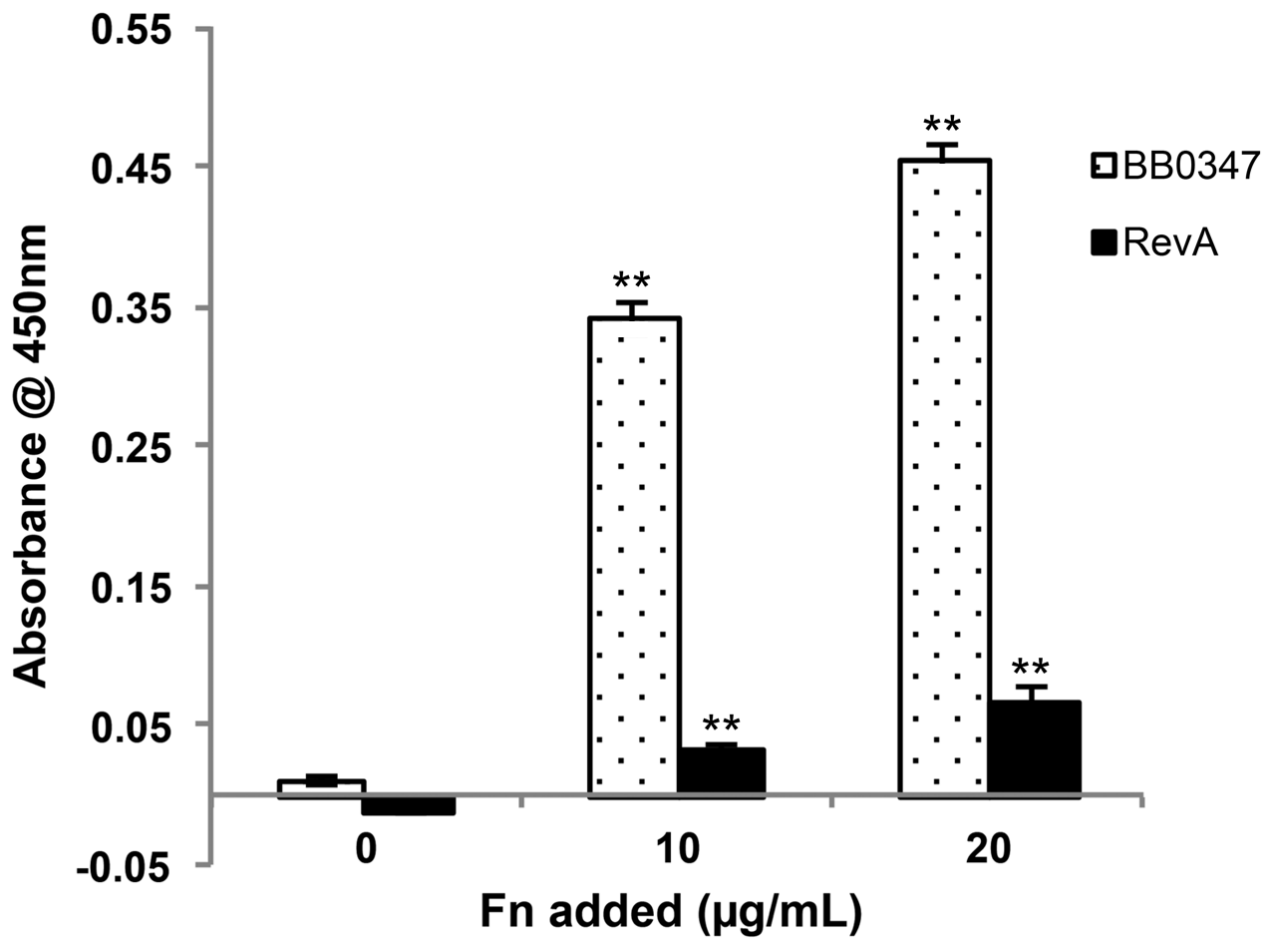
Recombinant BB0347 binds Fn in a dose-dependent manner. Fn-binding was determined by ELISA using BSA (negative control), RevA (positive control), and BB0347 as coating proteins. Fn was added to wells (0, 10, and 20 µg/mL) allowed to bind, and any unbound Fn was washed away. Values were blanked against the negative control to eliminate background from non-specific binding. BB0347 bound Fn in a dose-dependent manner as determined by an increased binding of αFn antibodies in wells treated with BB0347. Results are representative of three independent experiments–of at least 4 replicates for each concentration–with similar results, and error bars are indicative of ±SEM. Significance was determined by comparing the values of each concentration of Fn to the 0 Fn control values for both proteins after blanking with the BSA negative control.

After we verified that BB0347 is produced by infectious *B. burgdorferi*, our next objective was to determine whether or not the purified recombinant protein would be capable of directly binding Fn. Recombinant BB0347 and RevA (positive control) were coated to an ELISA plate as described above, and various levels of Fn were added to assess binding. Our rBB0347 bound the host protein in a dose-dependent manner (Fig. 2). This result supports the findings of a previous study in which surface plasmon resonance was used to quantify the binding between BB0347and Fn [21].

### Inhibition Assays

**Figure 3:**
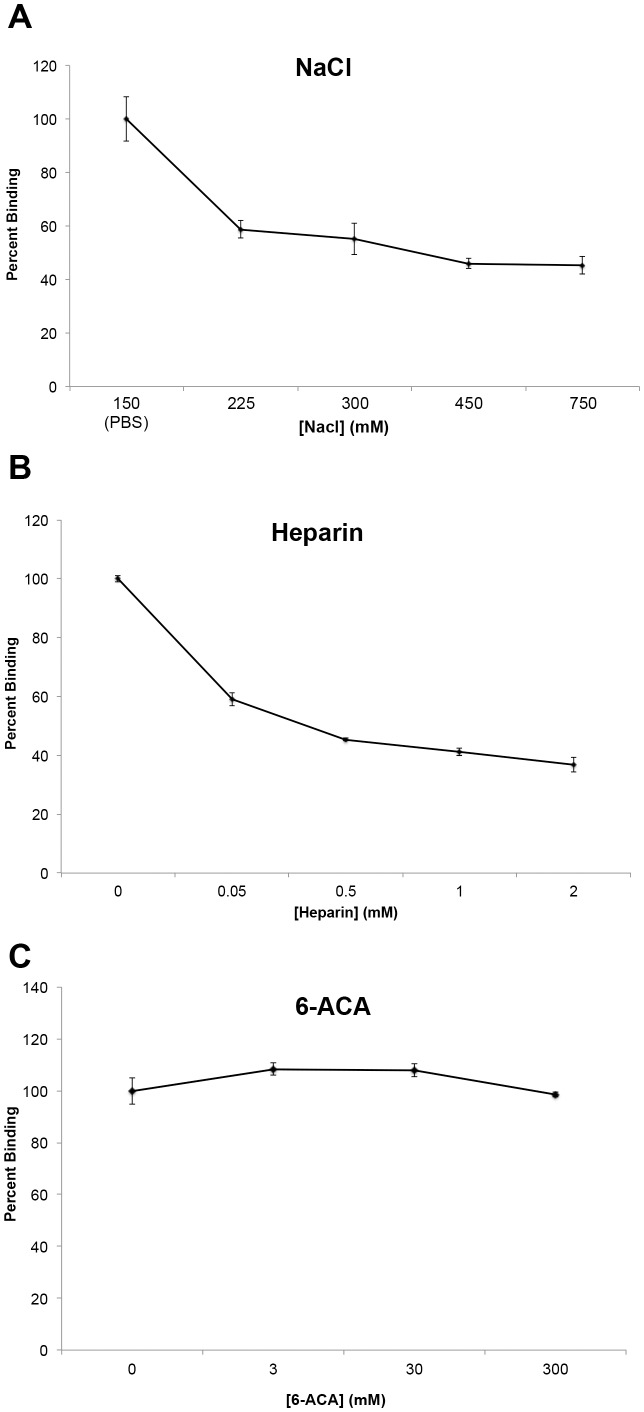
rBB0347-Fn binding is partially dependent on ionic interactions. BB0347 binding to Fn was measured via ELISA as in Fig. 2; except that varying levels of several inhibitors were used. Both NaCl (**A**) and heparin (**B**) inhibited the binding of BB0347 to Fn, while the addition of the lysine analogue 6-ACA (**C**) was not found to have any effect. All values were blanked against BSA. Results are representative of at least three independent experiments with similar results, and at least 4 replicates for each concentration. Error bars indicate ±SEM.

Since BB0347 has only recently been shown to directly bind Fn *in vitro*, the mechanisms of this binding are largely unknown. Previous studies have shown that the binding of Fn by several proteins is dependent on the ionic strength of the solution of incubation [49]–[51]. To determine if ionic interactions play a role in binding of BB0347 to Fn, a sodium chloride inhibition assay was performed (Fig. 3A). This assay works by releasing free ions into the buffer, thereby inhibiting interactions between potentially important charged residues on each protein. Increasing the NaCl concentration of the interaction buffer caused the binding between Fn and rBB0347 to decrease dramatically, implicating ionic forces from charged residues in the interaction of these two proteins. To verify these observations, we repeated the test with the physiological salt lithium heparin.

A heparin inhibition assay can work in two ways. Firstly, it is a negatively charged polysaccharide often found in the ECM, so it can function to inhibit ionic interactions like a NaCl inhibition assay [52]. Additionally, heparin is known to bind Fn, and can, therefore, also indicate if a given protein is competing at known heparin-binding sites [53]. We found that the addition of heparin inhibited the binding of Fn (Fig. 3B) by rBB0347, and that this inhibition was much more potent than that of NaCl (Fig. 3A). For example, binding was reduced by 40% after the addition of an estimated 50 µM heparin, while the same level of inhibition was not seen with the sodium chloride inhibition assay until a concentration of 75 mM NaCl (Fig. 3A). This indicates a more efficient form of inhibition occurs upon the addition of heparin to a BB0347-Fn binding phenomenon, even if the molar mass of heparin can only be estimated, as the difference in inhibition strength was approximately 1000-fold. However, our data also suggest that non-ionic (e.g. hydrophobic) interactions may also play a role in Fn-BB0347 binding, as we were unable to completely eliminate binding with either salt.

Additionally, many lysine residues are present in the primary structure of BB0347. As charged residues were implicated in BB0347-Fn binding, positively charged lysines could function as a source of these ionic interactions. To investigate this hypothesis, ε-aminocaproic acid (εACA, or 6-ACA) was added to the ELISA buffer in a manner similar to NaCl and heparin in the previous experiments. 6-ACA, as a lysine analogue, will competitively inhibit the activity of any lysine-recognizing residues in either protein [54], [55]. In our experiments, we were unable to find a role for lysine residues in the binding of rBB0347 to Fn, even upon the addition of up to 300 mM of 6-ACA (Fig. 3C). Additionally, we ensured that our protocol was effective and that the 6-ACA was still active by simultaneously using the inhibitor in a separate experiment to reduce the binding of borrelial enolase to plasminogen (Fig. S1) as described previously [54].

### Protease Treatment of Intact Spirochetes

**Figure 4:**
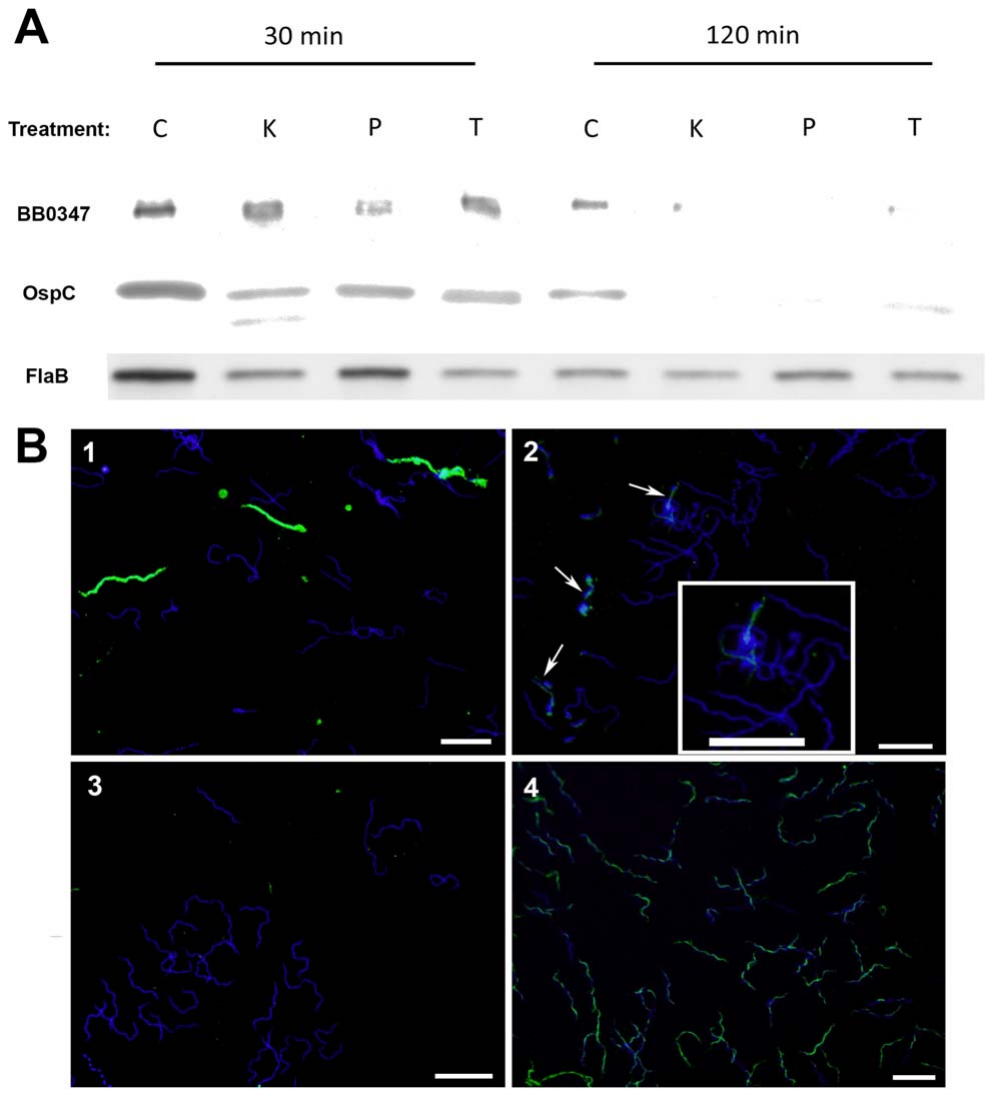
BB0347 is surface exposed in *B. burgdorferi* MI-16. **A)** Intact spirochetes were treated with different proteases for 30 min or 2 hrs. Western blots were run against whole cell lysates after these treatments and blotted with αBB0347, αOspC, or αFlaB. After two hours, no difference was observed between the control and protease-treated spirochetes in the αFlaB-blotted membranes, but OspC and BB0347 were almost completely degraded (Key: C: No protease control, K: proteinase K, P: pronase, T: trypsin). **B)** Intact bacteria were coated onto glass slides and fixed with 10% Formalin. Antibodies against the same proteins listed in (A) were used to stain and a secondary antibody conjugated with Dylight 488 was used for detection. DAPI was used as a secondary stain to localize spirochetes (see Materials and Methods). An additional control in which the *B. burgdorferi* membrane was compromised by desiccation, was included to verify that αFlaB antibodies were effective on fixed spirochetes. Key: Panel 1) αOspC, 2) αBB0347, 3) αFlaB 4) αFlaB with membrane disruption. BB0347 was detected in intact spirochetes, further verifying the surface exposure. White bars indicate a length of 10 µM, and the magnification is 1000×. Results are indicative of three independent experiments with similar outcomes.

In order for BB0347 to have a biological significance in live *B. burgdorferi*, it should be exposed on the outer membrane of the bacterium. A Triton X-114 solubility assessment [56] revealed that the protein was found to be associated with the membranes of the bacterium (data not shown), but did not allow for the exact subcellular location of the protein (e.g. the outer or inner membrane). To elucidate the exact subcellular location of BB0347, intact spirochetes were subjected to a protease treatment, as has been done to characterize borrelial outer membrane proteins previously [54], [56]–[58]. After treatment, a Western blot was performed against different borrelial proteins. FlaB was used as a loading and protease-resistant control because, in spirochetes, the flagella are located between the inner and outer membranes [59], [60], and degradation of FlaB would indicate that the outer membrane had been compromised. The proteases were able to digest BB0347 and OspC–a well-established borrelial surface marker [61]–[63] -almost completely after two hours, while FlaB levels remained constant in all of the two-hour lanes (Fig. 4A). These data suggest that BB0347 is located on the outer membrane of the gram-negative *B. burgdorferi* spirochete.

### Microscopy of Intact *Borrelia.*

To further verify that BB0347 is present on the outer membrane of *B. burgdorferi*, we also performed immunofluorescence microscopy with antibodies against the same three proteins used in the protease protection assay. This technique has also been used previously to verify the outer-membrane localization of proteins in spirochetes [64] as well as other microbes [55]. We found that *B. burgdorferi* presented heterogeneous expression of both OspC (12.1% of spirochetes were positive for OspC fluorescence) and BB0347 (8.9% positive), with more intense fluorescence emanating from the αOspC stained spirochetes (Fig. 4B). This is consistent when comparing the relative band intensity of the two proteins in the protease protection assay Western blots (αOspC lanes were exposed for 20 sec, while αBB0347 were exposed for 100 sec). αFlaB antibodies were again used as a negative control to ensure that the spirochetal outer membrane were intact, and, as expected, αFlaB produced no spirochete-specific staining in intact *B. burgdorferi*. To ensure that the lack of staining observed in the αFlaB samples was due to membrane impermeability and was not the result of poor antigen recognition of αFlaB *in situ*, we also included, as a control, spirochetes whose membranes were disrupted, then stained with αFlaB. These spirochetes all were shown to fluoresce, verifying that the αFlaB antibodies were effective. Therefore, the BB0347 was detected by immunofluorescence because it is surface exposed in intact spirochetes and not because of membrane-barrier subversion. Additionally, the percentage of OspC and BB0347-positive spirochetes did not increase upon membrane disruption, further supporting the hypothesis that BB0347 is entirely surface localized in *B. burgdorferi* MI-16.

### Localization of BB0347-Fn Binding

**Figure 5:**
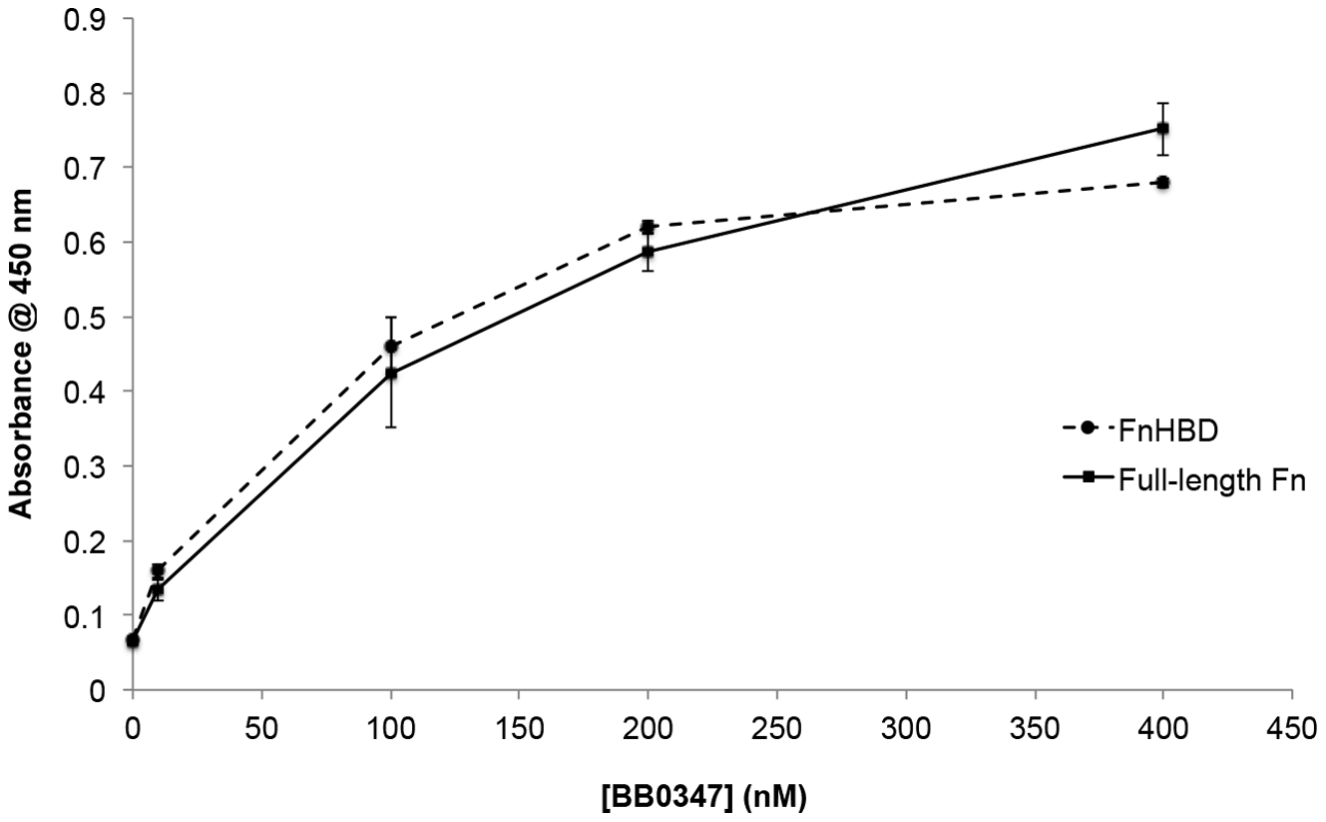
BB0347 interacts with the heparin-binding domain of Fn. An ELISA was performed analyzing the binding of recombinant BB0347 to both full-length Fn and the heparin-binding domain of Fn. A dose-dependent response in the binding of rBB0347 to the FnHBD was observed. No difference was observed between the interaction of BB0347 with either protein. The results are representative of three independent experiments with at least three replicates for every value. Error bars indicate ±SEM.

Fn has a variety of sites with which bacterial proteins might interact [16]. One such target is the Fn cell-binding domain (FnCBD), as has been observed in proteins from *Treponema pallidum*, such as TP0483 [42]. However, our observation that heparin strongly inhibited the binding of Fn by BB0347 (Fig. 3B), prompted examination of the binding between BB0347 and a peptide fragment containing the CS1 heparin-binding domain (HBD) of human Fn (Millipore, #F1903; Billerica, MA) via ELISA (Fig. 5). The KD values for both interactions were determined, and found to be similar (K_D_ rBB0347-Fn: 200±7 nm; rBB0347-FnHBD: 180±14 nm) indicating that this domain of the Fn molecule may be capable of being bound by BB0347 during an infection.

### Immunoreactivity of BB0347 in Mice

**Figure 6:**
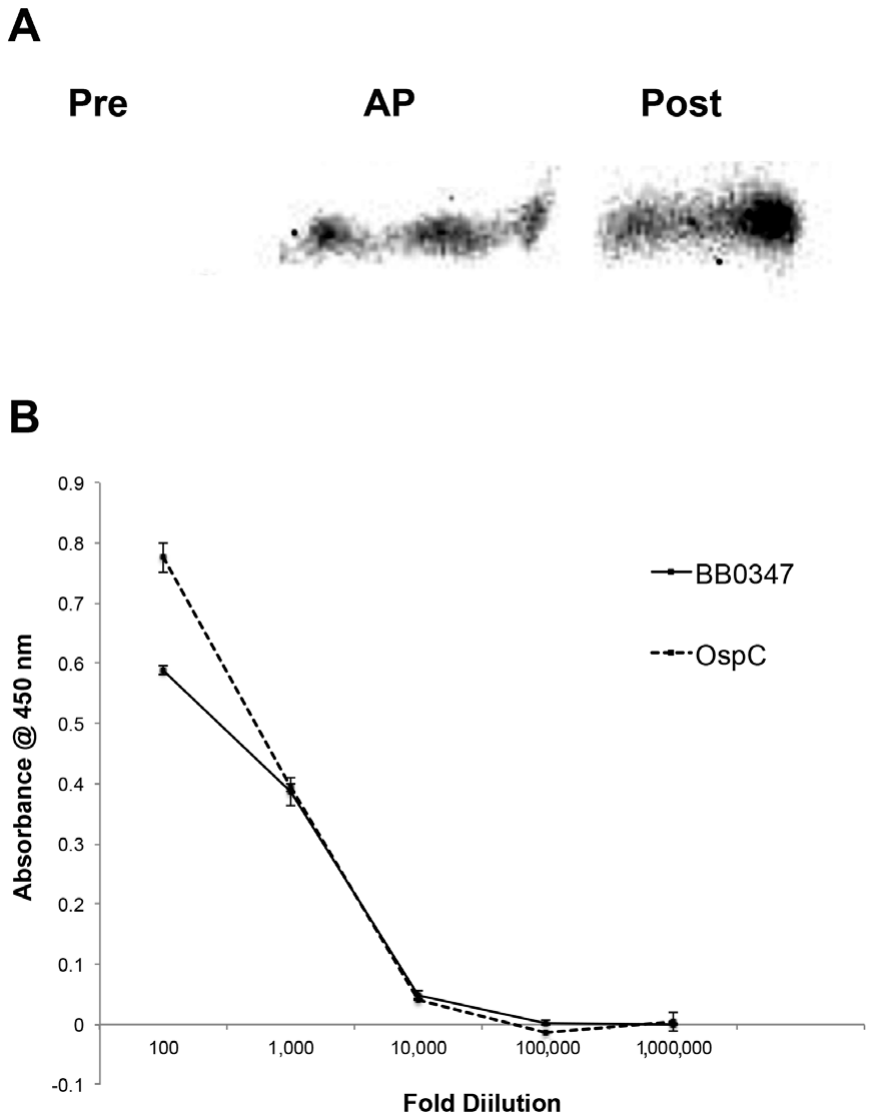
BB0347 is immunogenic in mice. **A)** Mice were inoculated with B. burgdorferi MI-16 and bleeds were collected before and 8 weeks after the injection of bacteria. Sera from four mice in each category were pooled for Western blotting against purified rBB0347. Lane 1) mouse pre-immune serum, Lane 2) affinity-purified αBB0347, Lane 3) mouse 8-week post infection serum. Bands of the appropriate size were observed in the positive control and infected mouse serum-treated lanes, but not the pre-immune serum treated lane. **B)** Antibody titers from the pooled sera were determined by ELISA. Values graphed are post-infected serum blanked for pre-infected. Antibodies responded to rBB0347 and OspC in the post-infected sera more strongly than those in the pre-infected sera up to a dilution of 1×10^−4^. Error bars indicate ±SEM, and data presented are indicative of three independent experiments with equivalent results.

To evaluate the potential for BB0347 as a target for therapeutics in a mammalian infection, we next determined the ability of mice infected with *B. burgdorferi* to produce antibodies that recognize recombinant BB0347. Sera collected from infected mice were able to recognize recombinant BB0347, whereas the collected pre-immune sera were not (Fig. 6A). This suggests a mounted adaptive immune response against BB0347 in a mouse infection model, and raises possibilities for the use of therapeutic agents that target BB0347. The same sera (pooled) were used to determine antibody titer levels through a ten-fold serial dilution ELISA. Antibodies were detected with a statistically higher response than those of uninfected serum to a maximum dilution of 1×10^−4^ (Fig. 6B). Antibodies against OspC were present at roughly the same level as those against BB0347, and both were detectable to the same dilution, although the initial response (before extensive dilution) was slightly higher in antibodies against OspC (Fig. 6B).

## Discussion

By using other well-researched pathogens as a model, we can infer that the binding of fibronectin may be an important phenomenon in borrelial pathogenesis [9], [11], [25], [65]. Additionally, the high level of redundancy in *B. burgdorferi* for Fn recognition [18]–[21] and phenotypes seen upon deletion of an Fn-binding protein [22], [23] further suggest that this host ECM protein may be important for the survival of the spirochete in a mammalian infection. Most work to date has been performed on the borrelial BBK32 protein, which was the first Fn-binding protein discovered in *B. burgdorferi*. However, a BLAST analysis of BB0347 revealed putative Fn-binding domains, and a recently published study found that BB0347 bound Fn via surface plasmon resonance [21]. This makes BB0347 the fifth protein thus far found in *B. burgdorferi* that is able to bind Fn–joining the ranks of BBK32 [18], RevA, RevB [19], and CRASP-1 [20].

Additionally, this study has been the first to confirm the expression and production of BB0347 by *B. burgdorferi* MI-16 in culture (Fig. 1A & B). We were also able to begin analysis of the optimal expression conditions for the *bb0347* mRNA. Our findings from this experiment suggest a role in mammalian pathogenesis as the production of BB0347 is regulated by the temperature of incubation, at least *in vitro* (Fig. 1C & D). This is consistent with many pathogenicity-associated genes from the bacterium [57], [66]–[68]. Additional work to characterize the conditions and effectors that control expression of BB0347 is underway.

It is important to note that is not within the scope of this study to compare the Fn-binding characteristics of RevA and BB0347, as has been previously published [21]. The differences between rRevA and rBB0347 observed in Figure 2 may not be accurate due to our coating conditions and available antibodies, and the only function RevA served was as a positive control for Fn-binding. However, once it was established that recombinant BB0347 binds Fn, the next objective was to begin characterizing the binding. BB0347 depends on ionic interactions for its full-strength interaction with Fn (Fig. 3A,B), thus implicating charged residues as important in the binding between the bacterial BB0347 and Fn, as is consistent with several other characterized bacterial proteins [49]–[51]. Conversely, another Fn-binding protein recently characterized in *B. burgdorferi–*RevA–has been shown to function independently of the buffer’s ionic strength [19], suggesting multiple mechanisms for bacterial interaction with this host protein. Furthermore, these data indicate that different Fn-binders may play different roles in borrelial pathogenesis due to their variation in interaction parameters. Despite the finding that ionic interactions are important, as well as the observation that high amounts of lysine residues are present in the sequence of BB0347, we were unable to find a role for that amino acid in BB0347-Fn binding via a 6-ACA inhibition assay (Fig. 3C). This finding suggests that lysines are either dispensable, or not important for direct interactions, although further work, such as site-directed mutagenesis of the lysine residues, is required to completely eliminate the possibility of lysine-dependent interactions.

The importance of BB0347 in an infection has not yet been established. In order for an ECM-binding protein to have biological significance, it should be presented on the outer membrane of the Lyme disease spirochete [54], [58], [64]. To verify that BB0347 is exposed to the outer surface of *B. burgdorferi*, we treated intact bacteria with several proteases: proteinase K, pronase, and trypsin. In all three treatments, the BB0347 and OspC were almost completely degraded, while the periplasmic FlaB exhibited no difference between the two-hour protease treatments and the no protease control (Fig. 4A). These data contradict the only other published study of BB0347/Fn interactions, in which the researchers were unable to detect the surface localization of that protein via a protease protection assay [21]. This is an interesting divergence that merits further investigation, and may be due to the fact that the BB0347 localization was previously determined in a strain overexpressing the protein, potentially altering BB0347’s transport within the spirochete.

Supplementing the protease digestion data for BB0347, immunofluorescence microscopy was used to view intact spirochetes (Fig. 4B). While OspC was present in far greater quantities on the spirochete surface (as evidenced by stronger fluorescence), BB0347 was still detectable on the *B. burgdorferi* membrane. Heterogeneity, a well-documented phenomenon in bacteria [69], [70] including in the *B. burgdorferi* OspC protein [61], [71], was observed in the production of both OspC and BB0347 (Panels 1 and 2). Our percentage of positive bacteria approximately matches those of the aforementioned studies. However, when analyzed with the protease protection data, these results suggest that the populations of bacteria actively producing OspC and BB0347 have completely localized these proteins to the outer membrane, which is also supported by the fact that disruption of the spirochete membrane had no effect on the staining pattern of either OspC or BB0347 (data not shown).

As previously stated, a potential function for a given Fn-binding protein could be the allowance of internalization of intact *B. burgdorferi* into non-phagocytic cells. This exact phenomenon has been observed recently in *B. burgdorferi* interactions with epithelial and fibroblast cells and has been shown to be dependent on β_1_ integrins; however, that study found no role for BBK32 in this internalization [35]. The question remains as to whether 1) a particular Fn-binding protein is important in this process; 2) several Fn-binders including BBK32 can fill in for each other (redundancy); or, 3) if one of the spirochete’s β_1_ integrin-binding proteins such as p66 or BapA [72] might facilitate this directly, as is known in other pathogens [17].

Determining the binding site for BB0347 on Fn may aid in the elucidation of a role for the interaction. For instance, a protein could cover the CBD to inhibit Fn-integrin-dependent signaling pathways [73]–or leave this domain available for potential internalization. We examined the accessibility of the FnCBD with the use of a CBD-specific antibody (provided by the University of Iowa DSHB), but the concentration provided was insufficient to confirm the effectiveness of our assay, even when a positive control for CBD-binding was used (rTP0483; data not shown). Further studies are currently underway to determine if the binding of Fn by BB0347 results in intracellular localization.

The observation that heparin inhibited the binding of rBB0347 to Fn (Fig. 3B) prompted the investigation of an FnHBD-BB0347 binding phenomenon. We found that an HBD-containing digest of Fn could bind with rBB0347 (Fig. 5). This region is distinct from the FnCBD and located in the CS1 domain of the eukaryotic protein [16], [21], [52]. Additionally, the binding curve and K_D_ value for the BB0347-FnHBD interaction closely resembled that of the borrelial protein with full-length Fn, thereby suggesting that this CS1 HBD may be the *in vivo* target of BB0347, as it is for Fn-binding adhesins from other bacteria [52], [74]. A CLUSTAL alignment of these two proteins with BB0347 did not reveal any domains of significant homology (data not shown), and the location of the potential binding site(s) for Fn on BB0347 merits further investigation.

Finally, we examined the potential for BB0347 to be targeted as a treatment for patients afflicted with Lyme disease, as well as a potential vaccine candidate. Immunogenicity of a molecule can, in some cases, directly translate to effectiveness of a vaccine targeting that molecule [75], [76]. Antibodies against BB0347 were detected in a sample of sera pooled from infected mice (Fig. 6A), indicating BB0347 as a potential target for immunotherapies, and suggesting that the protein is expressed during a mammalian infection. We also confirmed that our antibody recognized rBB0347 via an ELISA method (Fig. 6B), as we have done previously [43]. The reciprocal titer levels were comparable to measured levels against whole Borrelia lysates in infected wild animals [77], [78]. Further studies are needed to determine the immunogenicity of BB0347 in humans, as well as the prevalence and distribution of αBB0347 antibodies in an infected population of patients and the protective efficiency of the antibodies.

Further work is needed to elucidate the role of BB0347 in a murine infection. To this end, we are currently developing a deletion of *bb0347* in *B. burgdorferi*. Additionally, we are working to both determine the exact binding sites and any potential motifs for Fn binding in this protein as well as the mechanisms of control for the expression of the *bb0347* gene. Another interesting phenomenon is the high level of redundancy found in the proteins facilitating Fn binding in this bacterium. The reasons behind this redundancy remain unclear, and the question persists as to whether or not there is true redundancy or if each of the Fn-binding proteins from *B. burgdorferi* plays a unique role in the pathogenesis of the organism. Limited genetic tools for *B*. *burgdorferi* research make this a challenging question to answer, but by studying individual proteins we may begin to understand more about the infectious mechanisms of the Lyme disease spirochete.

## Supporting Information

Figure S1 **The 6-ACA lysine inhibition protocol is functional.** We ensured that the reagent and protocol was functional in reducing the binding of proteins dependent on lysines for interactions by interrupting the binding between borrelial enolase and plasminogen. BB0347-Fn interactions were still unaffected. Results are indicative of three independent experiments and error bars indicate ±SEM.
(TIF)
