# Supplementary figures and images for: BB0347, from the Lyme Disease Spirochete Borrelia burgdorferi, Is Surface Exposed and Interacts with the CS1 Heparin-Binding Domain of Human Fibronectin
Source: PLoS One. 2013 Sep 27;8(9):e75643. doi: 10.1371/journal.pone.0075643 (PMC3785480; doi:10.1371/journal.pone.0075643)

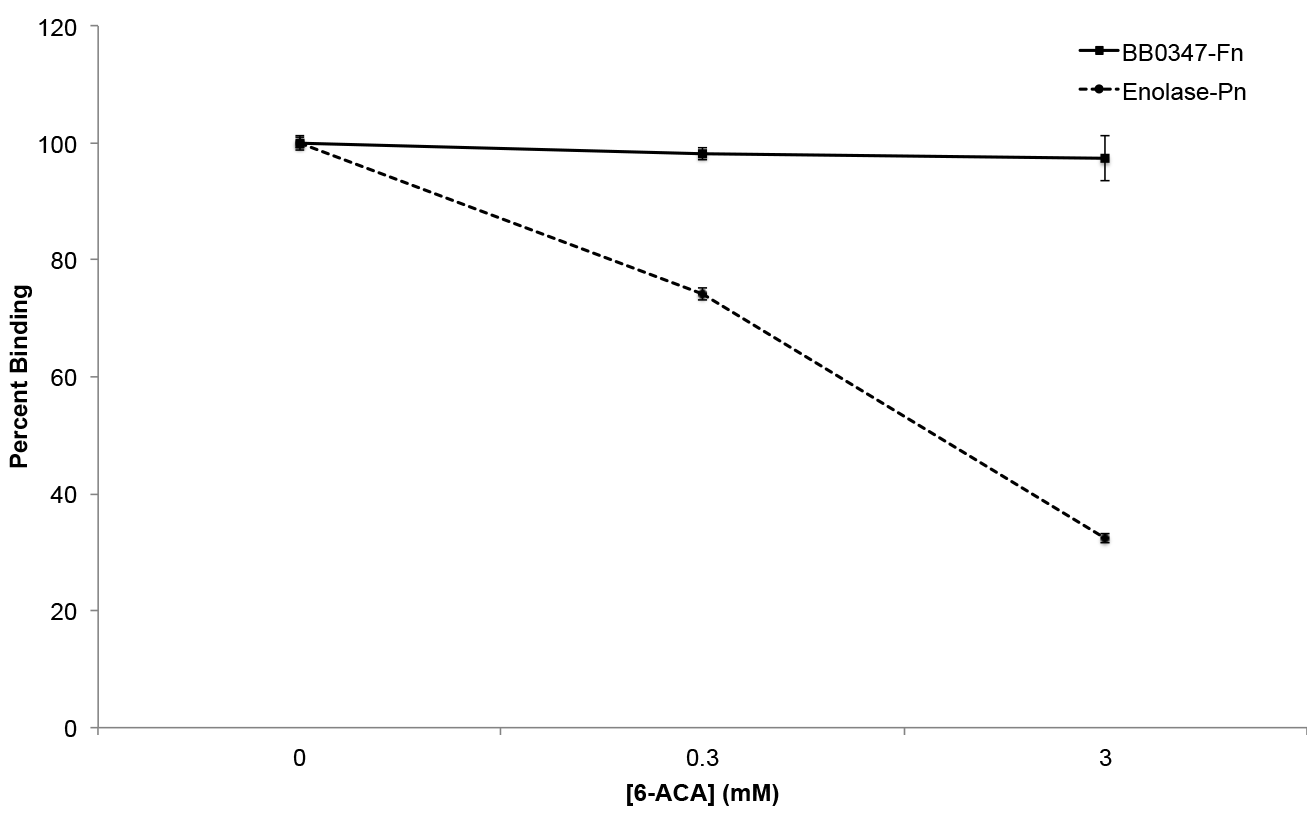

Supplement: Figure S1 — The 6-ACA lysine inhibition protocol is functional. We ensured that the reagent and protocol was functional in reducing the binding of proteins dependent on lysines for interactions by interrupting the binding between borrelial enolase and plasminogen. BB0347-Fn interactions were still unaffected. Results are indicative of three independent experiments and error bars indicate ±SEM. (TIF) [file pone.0075643.s001.tif]
